# Striatal and orbitofrontal grey matter volumes are associated with cognitive event related potentials during associative learning task performance in male and female children and adolescents

**DOI:** 10.3389/fnins.2026.1902749

**Published:** 2026-10-22

**Authors:** Kymbat Kassen, Rassul Shakhzadayev, Manzura Zholdassova, Oxana Kabenova, Altyngul Kamzanova, Gaukhar Datkhabayeva, Daniyar Abdilmanov, Jane B. Allendorfer, James Eliassen, Mikhail Ye. Mel’nikov, Almira Kustubayeva

**Affiliations:** 1 Department of Biophysics, Biomedicine, and Neuroscience, Farabi University, Almaty, Kazakhstan; 2 Brain Institute, Farabi University, Almaty, Kazakhstan; 3 University of Alabama at Birmingham, Birmingham, AL, United States; 4 University of Cincinnati, Cincinnati, OH, United States

**Keywords:** associative learning, event-related potentials (ERP), gray matter volume (GMV), N400, P300, sex differences, voxel-base morphometry

## Abstract

**Introduction:**

The individual differences in gray matter volumes and event-related potentials may underlie variability in associative learning. However, little is known about the sex differences in these structure–function relationships across development.

**Methods:**

The current study investigates associations between local gray matter volumes and brain responses during associative learning task in children and adolescents aged 7–21 years. We hypothesized that gray matter volumes of the basal ganglia and prefrontal cortex would be associated with event-related potentials indices of associative learning, in a sex-dependent manner. Final analysis included 138 participants (68 females, 70 males). Participants completed an associative learning task with concurrent EEG event-related potentials recording. Gray matter volumes were analyzed within 5 *a priori* regions of interest, predominantly located in the basal ganglia vicinity and prefrontal cortex.

**Results:**

In females, anterior N400 latency to Early-stage stimulus onsets was positively associated with gray matter volume in the left putamen/lentiform nucleus and to Late-stage stimulus onsets with gray matter volume in the subgenual anterior cingulate. Anterior P300 amplitude to Late-stage feedback presentation was positively associated with gray matter volume in the right nucleus accumbens/putamen and the right medial orbitofrontal cortex. Correlations involving the subgenual anterior cingulate and medial orbitofrontal cortex were significantly stronger in the female group relative to the male group. In males, anterior P300 amplitude to Early-stage feedback presentation was associated with gray matter volume in a left hippocampus/amygdala cluster, yet not significantly differently from females. In exploratory analysis, after accounting for total brain volumes, age was positively associated with the volumes of striatal and orbitofrontal regions in both sexes, with largely overlapping patterns.

**Discussion:**

Therefore, findings support structure–function relationships linking gray matter volumes of striatum and orbitofrontal cortex to brain activity during reinforcement learning performance. Given that associative learning is an instance of reinforcement learning, its underlying neural processes are influenced by the structural development of striatal and orbitofrontal regions.

## 1 Introduction

Individual differences in brain morphology constitute a neural basis for variability in cognitive abilities and behavior (Kanai and Rees, 2011; Takeuchi et al., 2017). Regional variations in gray matter organization are associated with attention, emotion, learning, and higher-order cognitive processes, suggesting that brain anatomy constrains functional processing and behavioral outcomes to some degree (Casey et al., 2005; Westlye et al., 2011; Okon-Singer et al., 2015; Takeuchi et al., 2017; Kustubayeva et al., 2026). Therefore, investigating brain morphology is critical for understanding individual differences in learning capacities and behavioral responses and offers a biologically grounded approach to linking neural architecture with cognitive function.

Voxel-based morphometry (VBM) is an effective method for studying local differences in gray matter volume (GMV), which allows for whole-brain, hypothesis-free analysis as well as for region-of-interest (ROI)-based approaches (Ashburner and Friston, 2000; Whitwell, 2009). The anatomical differences between groups or populations as revealed by VBM reflect the combined influence of genetic predisposition and experience-dependent plasticity across the lifespan, providing a window into the neural underpinnings of cognition, behavior and learning (Kanai and Rees, 2011; de Mendonça Filho et al., 2021).

Associative learning is a core cognitive process for establishing relationships between stimuli and outcomes, supporting prediction, adaptive behavior, and decision-making (Eliassen et al., 2012; Luque et al., 2017). This process involves two stages: an initial rule acquisition stage, characterized by higher demands on attention, working memory, and reasoning, followed by a rehearsal stage emphasizing memory stabilization and automation mechanisms (Eliassen et al., 2003). Associative learning relies on a distributed neural network involving temporal regions for associative representation and frontal areas for control and prediction (Hales and Brewer, 2011). However, being a type of reinforcement learning, associative learning primarily involves activity in the ventromedial prefrontal cortex and ventral striatum activity (Rodriguez et al., 2006). Notably, learning efficiency varies across individuals, suggesting that stable neural characteristics may contribute to differences in associative learning (Byrom, 2013).

Given that the cognitive processing of each stimulus-response-outcome triad unfolds dynamically over time, finely time-resolved measures such as event-related potentials (ERP) are well suited to capture its cognitive components (Zhang, 1998; Polich, 2007). While structural neuroimaging precisely quantifies anatomical characteristics, electrophysiology provides the advantage of defining the timing. Thus, a combination of voxel-based morphometry and ERPs links individual structural constraints to functional characteristics of different components of brain activity underlying associative learning processes (Qin et al., 2016; Kustubayeva et al., 2022).

The P300 is an ERP component that reflects higher-order cognitive processes related to directed attention, working memory, and decision-making (Polich, 2007). Typically emerging between 300 and 600 ms after stimulus onset, the P300 is elicited when stimuli are salient, task-relevant, or unexpected, and is commonly interpreted as an index of context updating and the allocation of cognitive resources (Polich, 2007; Brydges et al., 2020). The P300 component reflects activity within a distributed cortico-subcortical network, with major generators in the parietal cortex and modulatory influences from prefrontal, thalamic, basal ganglia regions supporting attentional allocation and context updating (Donchin and Coles, 1988).

The N400 ERP component, a negative wave occurring roughly 400 ms post-stimulus, reflects the integration of incoming stimuli into existing semantic, contextual, or associative frameworks and is traditionally linked to cortical semantic networks, particularly in temporal and frontal regions (Junge et al., 2021). Its modulation reflects the operation of cortico-subcortical systems involved in prediction, learning, and contextual updating (Hodapp and Rabovsky, 2021; Nour Eddine et al., 2024). Thus, N400 modulation can be understood as reflecting the outcome of interactions between cortical semantic representations and subcortical systems, rather than arising from cortical processes alone.

ERP components follow distinct developmental trajectories, reflecting maturation of sensory, semantic, and executive systems (Karakaş, 2024). Thus, N400 and P300 across development provides a temporally precise framework for understanding age-related changes in the neural underpinnings of attention, learning, and cognitive control (Riggins and Scott, 2020). Sexual differences in brain structure are also one of the reasons behind individual variability in cognitive development, and the development of certain brain areas responsible for reinforcement learning is subject to sexual differences in brain structure (Koolschijn and Crone, 2013). Therefore, sex-related variation should be similarly considered when examining ERP components (Gur and Gur, 2016).

Sex differences in ERP development may be partly explained by variability in the maturation of cortical and subcortical structures implicated in learning and cognitive control (Duerden et al., 2020). Indeed, structural MRI studies have demonstrated sex-dependent differences in the development of frontal, temporal, and subcortical regions, as well as in the connectivity within the fronto-striatal circuit (Spalek et al., 2023). Their structural properties may constrain the efficiency of neural processing indexed by ERP components, providing a potential anatomical basis for observed sex differences in electrophysiological markers of learning (Sumich et al., 2012). However, despite growing evidence that both brain structure and electrophysiological markers of learning exhibit sex-dependent developmental trajectories, there is still a lack of studies investigating structure-function relationships related to associative learning from the perspective of sex differences.

Therefore, the aim of the present study is to investigate the relationships between individual differences in brain structure, primarily in regions involved in associative learning, and electrophysiological markers of associative learning in male and female children, adolescents, and young adults.

## 2 Materials and methods

### 2.1 Participants

A total of 153 participants (mean age 14.89 (SD 3.96), age range 7–21, female = 75, male = 78) were recruited through word of mouth and advertisement. Participants were included in the study if they met the following criteria: ages 7–21, and of either sex (assigned at birth), were healthy and had no standard contraindications to MRI scanning, had normal or corrected-to-normal vision, were fluent in either Kazakh or Russian. The sample consisted of 73 Kazakh-speaking and 80 Russian-speaking individuals (based on their language of education at school). Exact age at the time of MRI acquisition, expressed in decimal years, was calculated based on participant’s date of birth and the date of the MRI scan.

The study protocol was approved by the Local Ethics Committee of the al-Farabi Kazakh National University (original approval IRB-A172, issued on September 10, 2020, and renewed by protocols IRB-A267 issued on February 22, 2021, and February 17, 2022). Written informed consent was obtained from all participants, and for minors, from their legal guardians, prior to participation. All procedures were conducted in full accordance with the principles of the 2013 revision of the WMA Declaration of Helsinki.

Sensitivity analyses were conducted using GPower 3.1 (Heinrich-Heine-Universität Düsseldorf, Germany; https://www.psychologie.hhu.de/arbeitsgruppen/allgemeine-psychologie-und-arbeitspsychologie/gpower). An analysis using the female sample, which was slightly smaller than the male sample, and assuming three regressors, one-tailed significance, *α* = 0.0125 (typical of small-volume–corrected *α* = 0.05), and a power of 0.80 demonstrated that the GLM analysis was adequately powered to detect effects with an effect size of *f*^2^ = 0.17, representing a moderate effect (Cohen, 1988). Additionally, an analysis assuming two-tailed *α* = 0.05 and power = 0.80 indicated that the correlation analysis with uncorrected significance level of *p* < 0.05 could detect results with an effect size of *r* ≈ 0.33, also reflecting a moderate-strength correlation (Cohen, 1988).

### 2.2 MRI and EEG acquisition parameters

Structural MRI data were acquired using a 3T GE SIGNA Architect scanner (GE Healthcare, Chicago, IL, USA) with a 3D T1-weighted BRAVO sequence. The acquisition parameters were as follows: repetition time (TR) = 7.716 ms, echo time (TE) = 3.14 ms, slice thickness = 1.2 mm, slice gap = 0.6 mm, and voxel size = 0.4688 × 0.4688 × 1.2 mm. A total of 260 sagittal slices were collected (matrix size = 512 × 512). Participants were instructed to remain still during scanning, and foam padding was used to minimize head motion. MRI-compatible headphones were provided for ear protection and noise reduction.

EEG data were recorded in a soundproof, electrically shielded room using an ANT Neuro eeGo amplifier with a 64-channel Ag/AgCl electrode cap positioned according to the 10–10 system while participants performed an associative learning task programmed in E-Prime. EEG data were initially recorded at a sampling rate of 4,096 Hz.

### 2.3 Associative learning task

A computer-based deterministic visual motor associative learning task was conducted, inspired by paradigm of Eliassen et al. (2003). In the experiment, subjects were required to respond to a stimulus appearing on either the left or right part of the screen by pushing the appropriate left or right arrow key. Subjects had to follow a rule which they had to discover through trial and error. This could have been “always left,” “always right,” “responding on the same side as the stimulus appears” or “responding on the opposite side from the stimulus.” A new rule would be introduced after every five to seven trials, signaled by a new picture for the stimulus.

Each trial started with a fixation cross lasting for 1.03 s, followed by the presentation of the stimulus for 2.25 s, where participants were supposed to respond. Any response taking more than this time period was regarded as erroneous. Then, after a delay of 0.53 s, the visual feedback about the correctness of the response (i.e., presentation of the word “Correct” or “Error” in the participant’s first language) was provided for 2 s. By using an efficient trial-and-error approach, the rule could be inferred in two exploratory trials making the third trial the first one with response made based on an already learned rule. This experiment included four blocks each containing six runs of 5 to 7 trials, thus consisting of a total of 192 trials.

Correct runs were defined as those for which there were no mistakes from the fourth trial on, while Incorrect runs were those where at least one mistake was made from the fourth trial on. To ensure task comprehension and sufficient attention to the task, only trials coming from the Correct runs were analyzed. The first three trials of every run were considered as part of the Early phase and referred to the process of learning the rule, while the fourth and following trials in the case of Correct runs were considered as part of the Late phase and referred to the use of the learned rule, resembling trials split into rule-acquisition and rehearsal phases by Eliassen et al. (2003). Behavioral data were extracted from E-Prime logs and synchronized with the EEG event file via a Python script. Behavioral variables were the percentage of correct responses (accuracy) and the average response time (RT) for correct trials on the Early and Late phases.

### 2.4 Gray matter volume computation and EEG processing

MRI data preprocessing was performed using SPM12 (Wellcome Centre for Human Neuroimaging, London, UK) running under MATLAB (MathWorks Inc., Natick, MA, USA). Raw DICOM data were converted to NIfTI format using the MRIConvert utility (Lewis Center for Neuroimaging, University of Oregon, USA). Tissue segmentation was performed in SPM12 to obtain probabilistic maps of gray matter, white matter, and cerebrospinal fluid in native space. Based on segmentation files, the per-tissue volumes were computed along with the total intracranial volume (TIV). Additionally, tissue class images were generated in rigidly aligned space for use in the Diffeomorphic Anatomical Registration Through Exponentiated Lie Algebra (DARTEL) utility (Ashburner, 2007).

To generate a study-specific anatomical template, DARTEL was applied to individual tissue maps. Following template creation, native-space GM images were spatially normalized into Montreal Neurological Institute (MNI) standard space using DARTEL flow fields and resampled to a voxel size of 1 × 1 × 1 mm^3^. The normalized modulated maps represent voxel-wise estimates of regional GMV, where signal intensity reflects the amount of tissue. The normalized modulated GM images were then smoothed with a Gaussian kernel of 2 mm full width at half maximum (FWHM).

EEG data were imported to EEGLab toolbox running under MATLAB, downsampled to 512 Hz and filtered with a 0.3–40 Hz bandpass finite impulse response filter. The channels with an average correlation with neighboring ones below 0.7 were interpolated, the EEG was re-referenced to the average, and subsequent Infomax independent component analysis (ICA)-based signal cleaning was performed. ICA components were classified using ICLabel version 1.6 and those that had a ≥88% chance of being Eye, Muscle or Channel Noise components were marked for exclusion. Then, the data were referenced to linked mastoid electrodes, and epochs were defined as –200 to 800 ms ranges relative to E-Prime-written event timestamps for stimulus onsets and feedbacks demonstration during the Early and Late stages of the learning. Baseline correction was performed using the –200 to 0 ms pre-event interval. Two sets of 9 leads each, namely, anterior (F3, Fz, F4, FC3, FCz, FC4, C3, Cz, and C4) and posterior (P3, Pz, P4, PO3, POz, PO4, O1, Oz, and O2) sets were defined for a subsequent analysis. Epochs exhibiting signal amplitudes >100 µV over 100-ms windows were excluded using a moving-window peak-to-peak procedure with a 25-ms step across the 18 channels of interest. The amplitudes and latencies of the N400 and P300 components were quantified separately for Early- and Late- stage stimulus-onset conditions, as well as for feedback-related epochs. The component time windows were determined based on visual inspection of grand-average ERPs. The curves were averaged within each event type, and peak amplitudes and latencies were extracted for P300 (300–450 ms) and N400 (300–500 ms), and then averaged across the channels included in each anterior or posterior set (see Figure 1). Peak amplitudes and latencies were first identified individually for each participant within the defined time windows; peaks located exactly at the lower or upper boundary of a time window were excluded. Fifteen participants produced fewer than 25 valid events in their largest category or fewer than 20 valid events in any category and therefore were excluded from further analysis. The final sample comprised 138 participants (68 females, 70 males), including 67 Kazakh-speaking and 71 Russian-speaking individuals mean age = 14.91 (SD = 3.98) years.

**Figure 1:**
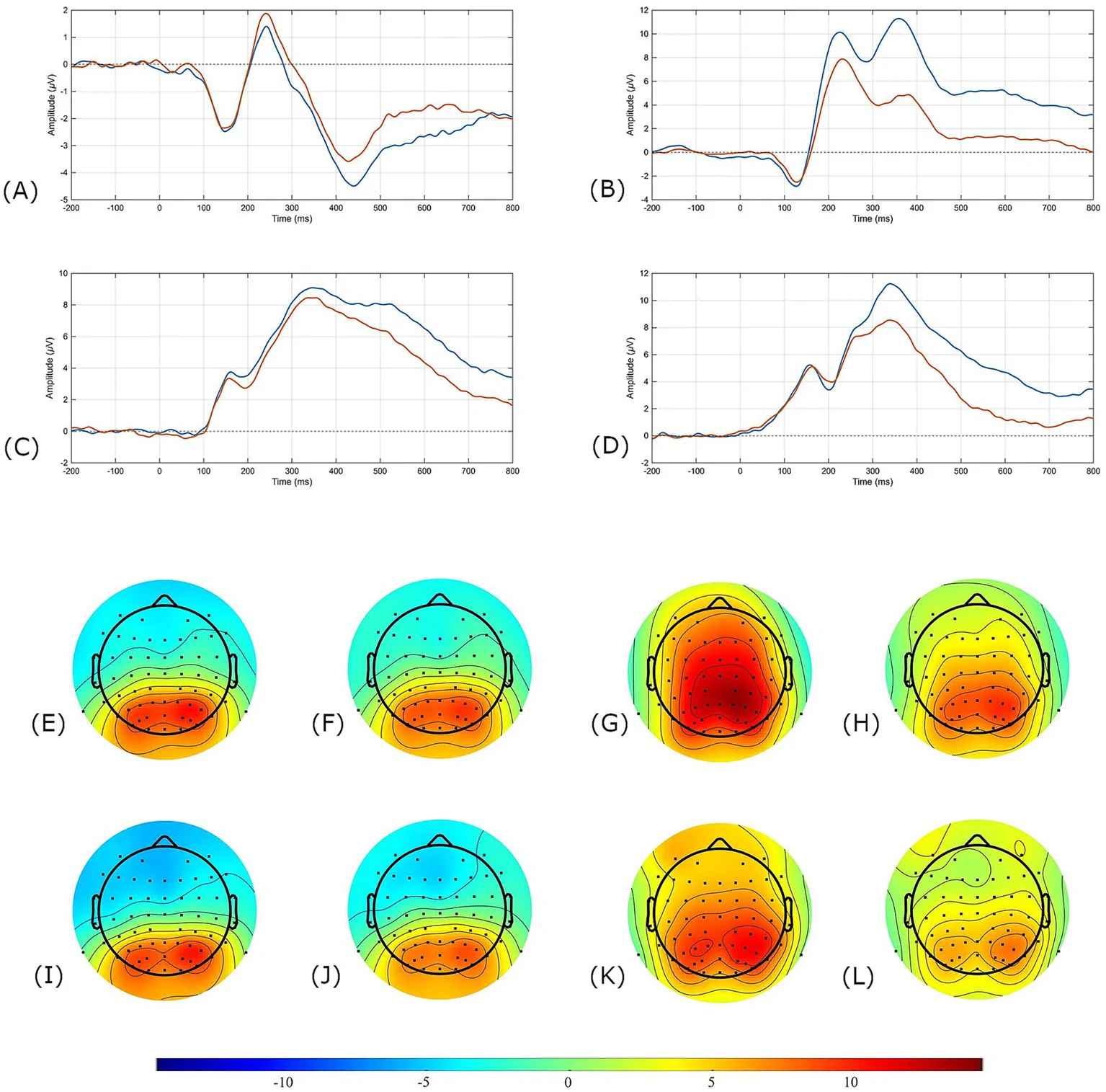
ERP time courses and topographies of the P300 and N400 components. **(A–D)** ERP time courses during the early and late associative learning task stages, relative to a cue stimulus onset at FCz **(A)** and POz **(B)** and relative to the feedback presentation at FCz **(C)** and POz **(D)**. Blue lines indicate the early stage; orange lines indicate the late stage. **(E–H)** ERP scalp topographies of the P300 during early and late associative learning task stages at 360 ms (averaged over 335–385 ms): **(E)** early-stage cue onset event, **(F)** late-stage cue onse event; **(G)** early-stage feedback presentation event, **(H)** late-stage feedback presentation event. **(I–L)** ERP scalp topographies of the N400 during early and late associative learning task stages at 435 ms, (averaged over 410–460 ms): **(I)** early-stage cue onset event, **(J)** late-stage cue onse event; **(K)** early-stage feedback presentation event, **(L)** late-stage feedback presentation event.

### 2.5 Statistical analysis

The collected data on RT were analyzed using two-way mixed analysis of variance (ANOVA), with time (Early, Late) as the within-participants factor, and sex (male, female) as the between-participants factor. The effect size was indicated by partial η^2^ values. Group differences in accuracy between female and male participants were assessed using a one-way ANOVA.

Statistical analyses of GMV were conducted using the general linear model (GLM) as implemented in SPM12. Modulated and spatially smoothed GM maps were entered into whole-brain voxel-wise multiple-regression analyses to investigate associations between regional GMV and participants’ ERP measures and age. In the main analysis, the ERP measures were used as regressors of interest while exact age, TIV, and primary language were included as covariates of no interest. Additionally, the constant term was modeled. Separate multiple-regression models were specified for each ERP value, assuming entering the relevant ERP measure (e.g., Early stage Onset-locked P300 amplitude over anterior EEG site) as regressors of interest to test for linear associations between ERP parameters and regional GMV. The one sample t-contrasts comparing the value with 0 were specified to assess positive and negative associations between GMV from one side and age or ERP regressors from the other side.

Statistical significance was assessed using a family-wise error (FWE)-corrected threshold of *p* < 0.05 at the voxel level within *a priori* regions of interest (ROI), i.e., small volume correction (SVC), with cluster threshold *k *≥ 10. The ROIs included 5 spheres with 6 mm radius, centered at local maxima of meta-analytical activation map at neurosynth.org generated for a term “reinforcement learning,” primarily in the striatum and ventromedial prefrontal cortex. The peaks coordinates (*x* *y* *z*) were the following for each ROI: 6 50 −4 (ROI 1, right orbitofrontal cortex, OFC); −8 6 −4 (ROI 2, left caudate nucleus); −14 2 −12 (ROI 3, left putamen); −14 6 −20 (ROI 4, left olfactory cortex); 10 4 −12 (ROI 5, right nucleus accumbens, NAcc) (see Figure 2). There was no additional multiple comparison correction implemented for the ROIs number or for ERP parameters number. Instead, the FWE correction was applied to correlation coefficients’ and their differences’ significance levels.

**Figure 2:**
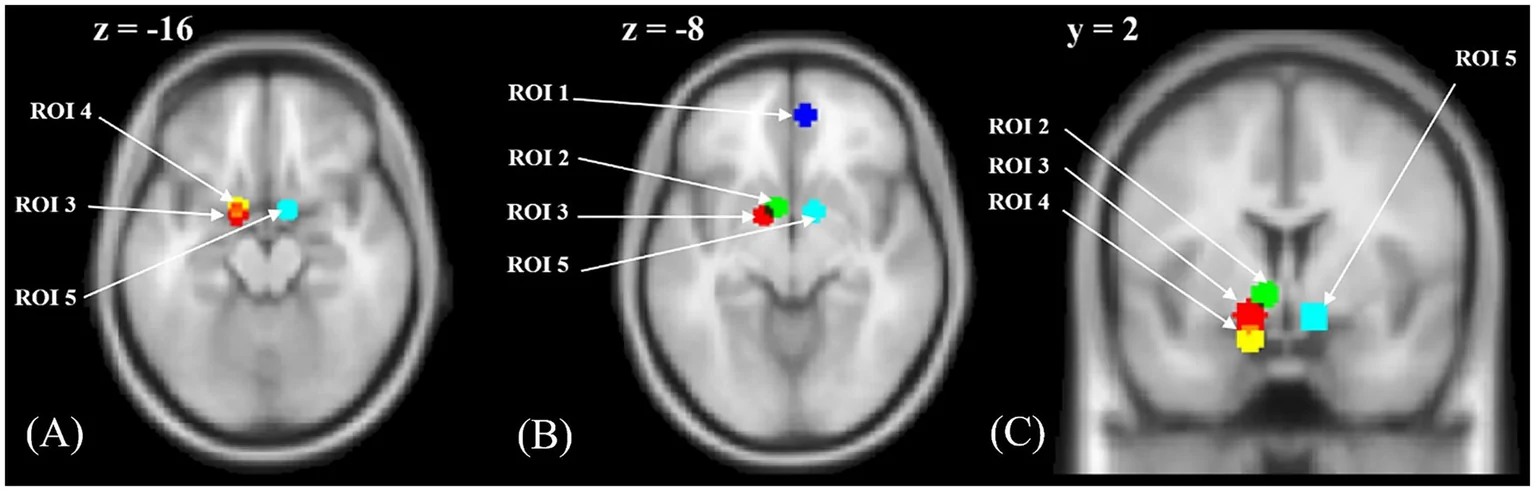
ROI positions. Each ROI corresponds to sphere with 6 mm radius derived from local maxima of meta-analytical statistical maps. Generated at neurosynth.org for a term “reinforcement learning”; ROI 1, right orbitofrontal cortex; ROI 2, left caudate nucleus; ROI 3, left putamen; ROI 4, left olfactory cortex; ROI 5, right nucleus accumbens; **(A–B)**: axial slices (*z* = −16 and *z* = −8). **(C)** Coronal slice (*y* = 22).

For subsequent correlation analysis, whitened and filtered voxel-wise GMV values were extracted and averaged within the identified cluster. In case of multiple clusters within one ROI, their values were averaged within each subject. Resulting averages and ERP parameter values were residualized for covariates, z-transformed, trimmed for outliers exceeding ±3 SD, and included in Pearson’s r correlation analyses with ERP measures similarly residualized for covariates and z-transformed. The confidence intervals were estimated using 1000 bootstrap iterations. The statistical significance of the correlation coefficients was adjusted using the FWE-correction for 80 analyses (5 ROIs × 2 ERP components × 2 sites × 2 features × 2 sexes). To compare the GMV−ERP associations between sexes, the mask of a cluster significant for one sex was applied to unthresholded statistical map of the other sex. The values extraction and correlation computation were performed exactly the same way. The correlation coefficients were then transformed using Fisher’s *r*-to-*z* transformation as *z* = arctanh(*r*), *Z* was calculated as:

$$Z=\frac{z_{1}-z_{2}}{\sqrt{\frac{1}{n_{1}-3}+\frac{1}{n_{2}-3}}}$$

and Φ(*Z*) using Fischer distribution. Last, corresponding two-tailed *p*-values were computed as 2 × (1 − Φ(*Z*)). These p-values were also adjusted using FWE-correction for 40 analyses (5 ROIs × 2 ERP components × 2 sites × 2 features). The correlation analyses were conducted using IBM SPSS 21.0 software.

The additional exploratory analysis for examining relationships between the local GMV and age followed the same general analytical procedure as that used for the ERP analyses, except ages of each participants’ were used as regressors of interest and ERP values were not included. However, given exploratory nature of the analysis, the statistical maps construction was not followed by the correlation analysis.

## 3 Results

### 3.1 Demographic data, ERP epoch counts, and tissue volumes in female and male groups

Demographic characteristics of the final analyzed sample are summarized in Table 1. The final sample consisted of 138 participants (68 females, 70 males). Male participants did not differ from females on mean age and on the ratio of Kazakh- and Russian-speaking individuals. Age distribution for the male and female groups did not significantly differ from a normal distribution (Kolmogorov-Smirnov’s *Z* = 0.92, *p* = 0.371 and *Z* = 1.07, *p* = 0.204 for male and female samples, respectively). Groups also did not differ significantly in a number of valid ERP epochs of any type averaged within-subject. Results in tissue volume characteristics revealed that male participants featured larger TIV and volume of each major tissue type, compared to female subjects.

**Table 1:** Demographic, tissue volume, and events count characteristics of the sample.

	Male (*N* = 70), *m* ± *σ*	Female (*N* = 68), *m* ± *σ*	*t*/*χ*^2^	*p*
Age	15.2 ± 3.8	14.7 ± 4.2	0.63	0.531
Language	33 Kazakh: 7 Russian	34 Kazakh:4 Russian	0.11	0.737
GM	0.86 ± 0.07	0.79 ± 0.07	6.65	<0.001
WM	0.45 ± 0.04	0.39 ± 0.04	7.59	<0.001
CSF	0.21 ± 0.06	0.18 ± 0.04	3.22	0.002
TIV	1.52 ± 0.11	1.36 ± 0.10	8.56	<0.001
Early onset-locked epochs	62.2 ± 18.6	57.8 ± 16.2	1.48	0.142
Late onset-locked epochs	61.3 ± 18.1	56.7 ± 16.6	1.57	0.118
Early feedback-locked epochs	60.7 ± 18.5	56.1 ± 16.8	1.50	0.136
Late feedback-locked epochs	60.8 ± 17.9	55.7 ± 15.7	1.79	0.076

*N*, sample size; *m*, mean; *σ*, standard deviation; *t*, Student’s *t*-test; *χ*^2^, Pearson’s chi-square test; *p*, two-tailed statistical significance level; GM, gray matter; WM, white matter; CSF, cerebrospinal fluid; TIV, total intracranial volume.

### 3.2 Associative learning task performance

Accuracy did not differ significantly between females (*M* = 0.695, SD = 0.196) and males (*M* = 0.723, SD = 0.203), *F*(1, 139) = 0.70, *p* = 0.404, *η*^2^_p_ = 0.01. The mean RTs (ms) were 756 (SD = 195) for males and 791 (SD = 161) for females in the Early condition, and 632 (SD = 169) for males and 662 (SD = 161) for females in the Late condition. RTs were significantly slower in Early than in Late trials (*F*(1, 135) = 238.6, *p* < 0.001, *η*^2^_p_ = 0.64). There was no significant effects of sex (*F*(1, 135) = 1.33, *p* = 0.25, *η*^2^_p_ = 0.01) or an interaction between time and sex factors (F(1, 135) = 0.07, *p* = 0.79, *η*^2^_p_ = 0.00).

### 3.3 ERP results

ERP results are reported below separately for onset-locked and feedback-locked components (see Table 2 for a summary). In females, the onset-related anterior N400 latency on the Early task stage was positively associated with GMV within ROI 3 (*r* = 0.437, *p*_unc_ < 0.001, *p*_FWE_ = 0.014, CI [0.203, −0.619]), with two clusters encompassing the left putamen extending into the lentiform nucleus. By contrast, no significant association was observed in males (*r* = –0.122, *p*_unc_ = 0.315, *p*_FWE_ = 1.0, CI [−0.328, 0.124]). The difference between correlations yet presented a trend approaching significance level was not significant (*Z* = 3.2, *p*_unc_ = 0.001, *p*_FWE_ = 0.050). One more positive association was found in female group between GMV cluster in the ROI5 and the N400 latency, located in the extra-nuclear white matter (see Table 2). Despite the GMV-ERP correlation was significant in female group (*r* = 0.441, *p*_unc_ < 0.001, *p*_FWE_ = 0.012, CI [0.247, 0.605]) and insignificant in male group (*r* = −0.043, *p*_unc_ = 0.724, *p*_FWE_ = 1.0, CI [−0.269, 0.143]), the inter-group difference was not significant (*Z* = 2.8, *p*_unc_ = 0.005, *p*_FWE_ = 0.207).

**Table 2:** ERP-associated GMV results in *a priori* ROIs.

*K*	MNI			Peak *t*	Main regions	# ROI
	*x*	*y*	*z*			
Females (*N* = 68)						
**Anterior N400 latency to early onset**						
16	11	−1	−10	3.78	Extra-nuclear*	5
27	−17	4	−8	4.01	L. putamen, lentiform nucleus	3
14	−13	1	−12	3.44	L. putamen, lentiform nucleus	3
**Anterior N400 latency to late onset**						
32	−3	2	−6	4.56	L. anterior cingulate, subgenual, extra-nuclear*	2
**Anterior P300 amplitude to late feedback**						
96	12	6	−8	4.28	Extra-nuclear*, R. nucleus accumbens, lentiform nucleus, putamen	5
21	4	54	−5	3.82	R. medial orbitofrontal cortex, medial frontal gyrus	1
**Anterior P300 latency to late feedback**						
12	−3	2	−5	−3.79	L. anterior cingulate, subgenual	2
Males (*N* = 70)						
**Anterior N400 amplitude to early onset**						
13	−11	0	−10	3.50	Extra-nuclear*	3
**Anterior N400 amplitude to late onset**						
12	−5	4	−1	−3.81	L. Caudate nucleus	2
**Anterior P300 amplitude to early feedback**						
27	−17	−3	−10	4.34	Extra-nuclear*, L. hippocampus, L. amygdala	3
**Posterior P300 amplitude to early onset**						
25	−11	1	−9	4.53	Extra-nuclear*	3

*k*, number of voxel within a cluster; MNI, coordinates in the Montreal Neurological Institute space; *t*, Student’s *t*-test; # ROI, region of interest (for position, see Figure 2); R., L., right or left hemisphere. *The cluster was located in the extra-nuclear white matter region.

Additionally, in female group, the latency of Late-stage onset-related anterior N400 was positively associated with GMV within ROI 2 (*r* = 0.486, *p*_unc_ < 0.001, *p*_FWE_ = 0.002, CI [0.274, 0.662]), specifically in the subgenual anterior cingulate (sgACC). No significant association was observed in males (*r* = –0.107, *p*_unc_ = 0.378, *p*_FWE_ = 1.0, CI [−0.354, 0.191]). The difference between correlations was significant (*Z* = 3.4, *p*_unc_ < 0.001, *p*_FWE_ = 0.025) (see Figure 3).

**Figure 3:**
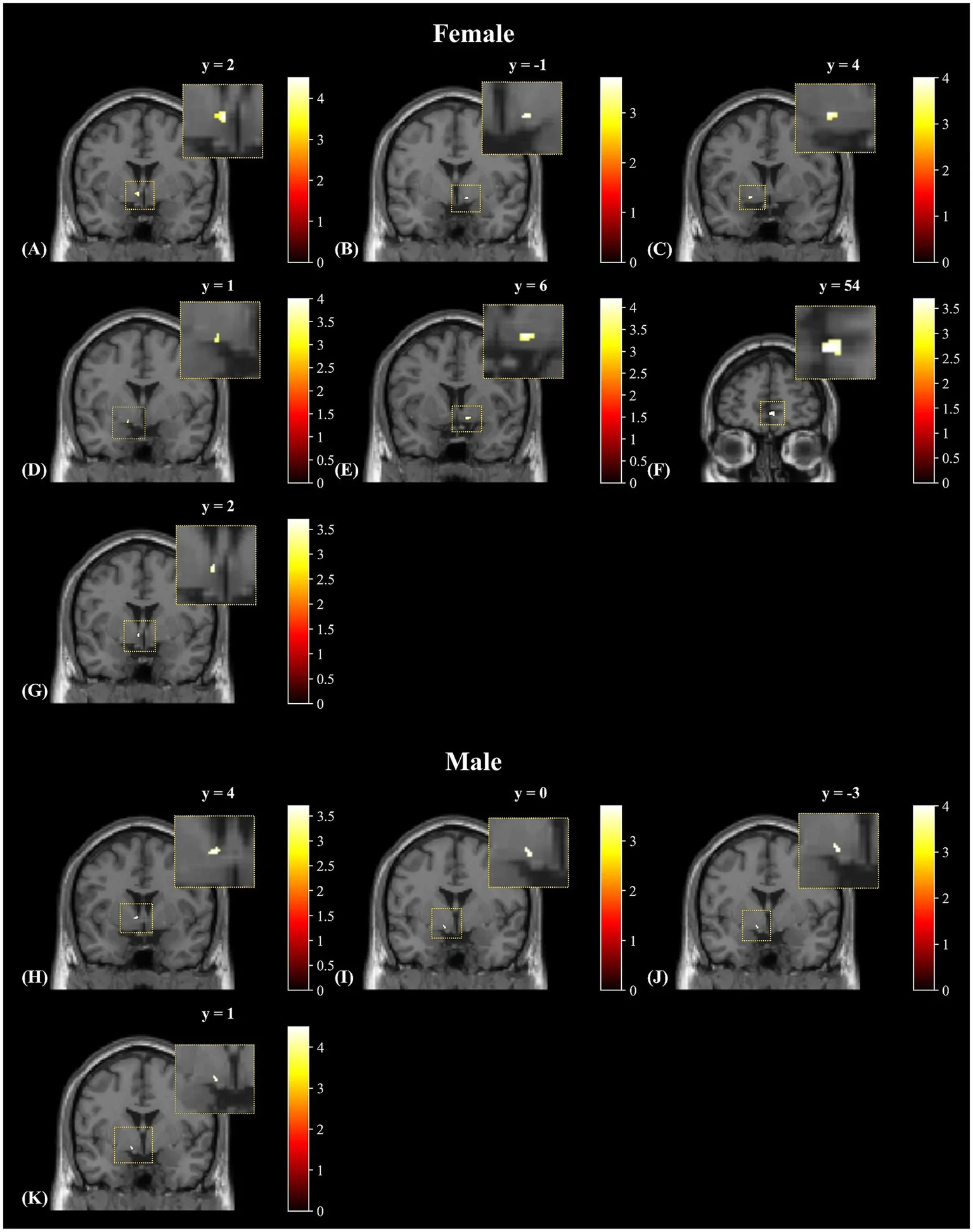
The clusters of GMV within each *a priori* ROI that is significantly associated with ERP properties. **(A)** Female group, GMV associated with anterior N400 latency to Early Onset in ROI 5. **(B)** Female group, GMV associated with anterior N400 latency to Early Onset in ROI 3. **(C)** Female group, GMV associated with anterior N400 latency to Early Onset in ROI 3. **(D)** Female group, GMV associated with anterior N400 latency to Late Onset in ROI 2. **(E)** Female group, GMV associated with anterior P300 amplitude to Late Feedback in ROI 5. **(F)** Female group, GMV associated with anterior P300 amplitude to Late Feedback in ROI 1. **(G)** Female group, GMV associated with anterior P300 latency to Late Feedback in ROI 2. **(H)** Male group, GMV associated with anterior N400 amplitude to Early Onset in ROI 3. **(I)** Male group, GMV associated with anterior N400 amplitude to Late Onset in ROI 2. **(J)** Male group, GMV associated with anterior P300 amplitude to Early Feedback in ROI 3. **(K)** Male group, GMV associated with posterior P300 amplitude to Early Onset in ROI 3. y is the position of a slice in MNI coordinate system. All results significant at *p* < 0.05 at voxel level, SVC corrected. Scales indicate *t*-value.

On the Late learning stage, feedback-locked anterior P300 amplitude showed positive associations with GMV in two clusters. The first cluster was located within ROI 5, in the right NAcc, lentiform nucleus, and putamen, with some extra-nuclear white matter. The female group demonstrated positive ERP-GMV correlation (*r* = 0.461, *p*_unc_ < 0.001, *p*_FWE_ = 0.005, CI [0.241, 0.653]). No significant association was observed in males (*r* = 0.216, *p*_unc_ = 0.072, *p*_FWE_ = 1.0, CI [−0.067, 0.451]), however the difference between two groups in correlation strength was not significant (*Z* = 1.4, *p*_unc_ = 0.159, *p*_FWE_ = 1.0). The second cluster was identified in females within ROI 1 in the right medial OFC (MOFC, *r* = 0.465, *p*_unc_ < 0.001, *p*_FWE_ = 0.005, CI [0.224, 0.660]). In males, the corresponding association was negative and insignificant (*r* = −0.237, *p*_unc_ = 0.048, *p_F_*_WE_ = 1.0, CI [−0.427, −0.031]). The difference between the correlation coefficients was significant (*Z* = 4.0, *p*_unc_ < 0.001, *p*_FWE_ = 0.002).

On the Late learning stage, anterior P300 latency in response to feedback showed a negative association with GMV in females within ROI 2 (*r* = −0.434, *p*_unc_ < 0.001, *p*_FWE_ = 0.016, CI [−0.633, −0.199]), with the cluster located in the right sgACC. In males, no significant association was observed within the corresponding region (*r* = −0.006, *p*_unc_ = 0.963, *p*_FWE_ = 1.0, CI [−0.258, 0.214]). The difference between correlation coefficients did not survive FWE correction (*Z* = −2.5, *p*_unc_ = 0.014, *p*_FWE_ = 0.530).

In males, the stimulus onset-locked anterior N400 amplitude produced positive association cluster on the Early stage in the extra nuclear white matter (*r* = 0.422, *p*_unc_ < 0.001, *p*_FWE_ = 0.020, CI [0.276, 0.608]) and negative association cluster on the Late stage in the left caudate (*r* = −0.425, *p*_unc_ < 0.001, *p*_FWE_ = 0.017, CI [−0.593, −0.181]) (see Table 2). Both correlations were not significant in females (*r* = −0.018, *p*_unc_ = 0.886, *p*_FWE_ = 1.0, CI [−0.289, 0.252] and *r* = 0.058, *p*_unc_ = 0.638, *p*_FWE_ = 1.0, CI [−0.192, 0.276] respectively), while sex differneces in correlation strength were also non-significant (*Z* = 2.5, *p*_unc_ = 0.012, *p*_FWE_ = 0.439 and *Z* = −2.8, *p*_unc_ = 0.006, *p*_FWE_ = 0.211, respectively).

In the male group, the anterior P300 amplitude in response to feedback on the Early task stage showed a positive association with GMV within ROI 3 in a cluster including the left hippocampus and amygdala. In males, the correlation was significant (*r* = 0.481, *p*_unc_ < 0.001, *p*_FWE_ = 0.002, CI [0.293, 0.642]). In contrast, no significant association was observed in females (*r* = 0.079, *p*_unc_ = 0.522, *p*_FWE_ = 1.0, CI [0.642, 0.311]). The difference between the male and female groups’ correlations was not significant (*Z* = 2.3, *p*_unc_ = 0.021, *p*_FWE_ = 0.796). An additional cluster of GMV located in the left extra-nuclear white matter associated with the posterior P300 amplitude to Early-stage onset-locked events was revealed (see Table 2). It produced a significant correlation in the male sample (*r* = 0.484, *p*_unc_ < 0.001, *p*_FWE_ = 0.002, CI [0.279, 0.636]) and an insignificant correlation in the female sample (*r* = 0.199, *p*_unc_ = 0.106, *p*_FWE_ = 1.0, CI [−0.014, 0.385]), though inter-group difference in correlation strength was not significant (*Z* = 1.6, *p*_unc_ = 0.103, *p*_FWE_ = 1.0).

### 3.4 Exploratory analysis: age-related results

Male and female groups demonstrated broadly similar positive associations between the age and GMV within five *a priori* ROIs. Age-related GMV maps comprised volumes of several prefrontal, striatal, limbic, and olfactory regions in both groups (Table 3). Within ROI 1, both females and males showed age-related GMV effects in right medial frontal regions, including MOFC, medial frontal gyrus, and ACC regions. In ROI 2, effects of age for both groups were concentrated in the left striatum, specifically in the caudate, putamen and pallidum, while for females effects also involved the NAcc. In ROI 3, both groups had effects in the left olfactory cortex, putamen and amygdala, while the NAcc was additionally involved for males. Within ROI 4, both males and females had age effects in the left olfactory and left postero-medial OFC, whereas males demonstrated additional effects in medial and inferior frontal gyri areas. Finally, in ROI 5, females and males had similarly localized age effects in the right NAcc and olfactory cortex.

**Table 3:** Age-associated GMV results in *a priori* ROIs.

*K*	MNI			Peak *t*	Main regions	# ROI
	*x*	*y*	*z*			
Females (*N* = 68)						
740	4	45	−7	6.76	R. medial orbitofrontal cortex, R. medial frontal gyrus, R. anterior cingulate cortex, R. superior medial frontal cortex	1
795	−12	4	−8	10.74	L. nucleus accumbens, L. pallidum, L. caudate nucleus, L. putamen	2
683	−13	4	−8	11.47	L. olfactory cortex, L. putamen, L. amygdala	3
401	−18	5	−16	9.36	L. olfactory cortex, L. posterior orbitofrontal cortex, L. medial orbitofrontal cortex	4
685	13	5	−9	9.00	R. nucleus accumbens, R. olfactory cortex	5
Males (*N* = 70)						
825	7	53	−5	6.88	R. medial orbitofrontal cortex, R. medial frontal gyrus, R. superior medial frontal cortex, R. anterior cingulate cortex	1
743	−10	7	−6	7.42	L. pallidum, L. caudate nucleus, L. putamen	2
677	−14	4	−8	7.77	L. olfactory cortex, L. putamen, L. amygdala, L. nucleus accumbens	3
423	−15	6	−17	6.88	L. olfactory cortex, L. subcallosal gyrus, L. medial frontal gyrus, L. inferior frontal gyrus, L. posterior orbitofrontal cortex, L. medial orbitofrontal cortex	4
674	7	−1	−10	7.45	R. nucleus accumbens, R. olfactory cortex	5

*k,* number of voxel within a cluster; MNI, coordinates in the Montreal Neurological Institute space; *t,* Student’s *t*-test; # ROI, region of interest (for position, see Figure 2); R., L., right or left hemisphere.

## 4 Discussion

In the current study, long-latency ERPs recorded during associative learning task were utilized to measure properties of cognitive brain activity, while VBM assessed regional GMV. Correlating these parameters separately in male and female groups and comparing the correlation coefficients enabled investigation of sex-related structure-function associations in the associative learning function development.

The ERP−VBM analysis found that the structural-functional associations indeed differed between sexes. In female participants, the anterior N400 latency on the Early and Late task stages was found to be correlated with GMV, predominantly in striatal and ACC regions, respectively. The Late stage-related positive association pattern differs significantly from the one found in the male group. P300 during the Late-phase feedback presentation was associated with GMV in the ventral striatum, OFC (P300 amplitude) and sgACC areas (P300 latency). The OFC association significantly differed from that in the males group.

In male participants, the onset-locked anterior N400 amplitude on the Early and Late task stages was found to be associated with GMV in the extra-nuclear region and caudate nucleus, respectively. The anterior P300 amplitude in response to feedback presentation in the Early learning period was correlated with GMV in the medial temporal area, specifically in the amygdalar/hippocampal area. Last, onset-locked posterior P300 amplitude on the Early task stage was associated with the local GMV in the extra-nuclear white matter.

Of note, the associations located in the extra-nuclear area probably reflect individual differences in the basal ganglia volume or shape. Morphological variation in larger neighboring GM structures may extend into voxels classified by the anatomical atlas as extra-nuclear white matter. Therefore, these findings may reflect morphological differences in adjacent GM structures rather than effects related to the extra-nuclear white matter area per se.

### 4.1 Gray matter volume, sex differences and associations with age

There were no differences in average age and primary language ratio between males and females in this study. However, groups differed in terms of TIV and the volume of each tissue type, evidencing larger brain volumes in male participants that aligns well with prior studies (Gur et al., 1999). The significant difference in the TIV and especially in the total GMV further motivate separate analyses of male and female groups and between-group comparisons.

The exploratory analysis demonstrated that, after accounting for the global age effects on the brain structure, both groups exhibited GMV clusters within the five *a priori* ROIs featuring significant positive associations with the age of the subjects. Age and GMV associations were observed in such regions as the medial prefrontal/OFC region, olfactory cortex, limbic system, and the subcortical nuclei (amygdala, NAcc, pallidum, putamen, and caudate nucleus). Although previous research have described an overall global decrease in GMV with age (Gennatas et al., 2017), our results imply differential structural maturation of the mentioned regions, consistent with previous findings (Goddings et al., 2014; Gennatas et al., 2017). However, because these analyses were exploratory, the observed regional associations should be interpreted cautiously and warrant replication in larger, independent samples. An additional reason for caution in such interpretations is the VBM technique offers only an indirect measure of brain morphology (Gur et al., 1999; Nopoulos et al., 2000; Lotze et al., 2019).

### 4.2 Neural correlates of N400 in associative learning

The pattern of N400–GMV associations differed descriptively between females and males; however, not all between-sex comparisons were statistically significant. In females, during the Early learning stage, longer onset-related anterior N400 latency was significantly associated with GMV in the left putamen/lentiform nucleus; yet this association did not differ significantly from that observed in males. This link is broadly compatible with the established role of the dorsal striatum, particularly the putamen, in stimulus-response learning and the acquisition of habitual action patterns (Hikida et al., 2017; Munding et al., 2025; Haruno and Kawato, 2006), crucial for the associative learning. Indeed, stimulus onset, regardless of the task stage requires mapping the stimulus to motor action considered matching the stimulus, and the Early stage also incorporates the update of the learned stimulus-response mapping, making this structure-function correlation logical.

Importantly, in female participants, Late-stage onset-locked anterior N400 latency was associated with GMV in the left ACC region. The sgACC is involved in assessing the motivational and emotional context (Joyce et al., 2023). In the present associative learning paradigm, this relationship may involve neural processes involved in integrating affective and motivational context during the processing of learned stimulus-outcome associations at later stages of learning. Interestingly, this association occurred with onset-related N400, not with feedback-related ERP measure, that might be more directly related to eliciting emotional reactions via feedback. It is possible though that the link reflects the influence of the feedback preserved to the point of making subsequent choice. Note that the Late-stage feedback could be positive only in the analyzed trials and instead of signaling right or wrong response it rather demonstrated that the deduced rule is still active and was understood correctly. In women, the relationship between the temporal dynamics of contextual processing and sgACC GMV was significant, whereas no significant relation was found among men. The relationship also showed significant difference between the sexes. Such a pattern could be indicative of sexually dimorphic organization of the neural networks underlying sgACC (Osborne et al., 2021), in which sgACC structure would be more associated with contextual processing dynamics in females, though more research is warranted in this area.

A different late-stage N400-related structure–function pattern was observed in males. Late-stage onset-locked N400 negative amplitudes in male participants showed a positive association with GMV in the caudate. The caudate is also related to the reward anticipation (Knutson and Greer, 2008), that matches the fact that in the current task on the Late stage of analyzed trials the feedback could be only positive. Additionally, the caudate nucleus is involved in action selection and learned action–outcome contingencies (Balleine et al., 2007). In fact, the establishment of the stimulus-response-outcome mapping is shared between the putamen, demonstrating the significant structure-function relationships in the female sample and the caudate involved in structure-function relationships in males. Given, there are no significant differences in correlation strength between these groups, studies with larger samples are required to define whether these links encompassing two functionally connected basal nuclei are actually sex-specific or represent parts of a single neural associations, differentially highlighted in our study.

The N400 is primarily considered a semantic-related ERP component and has been studied extensively in verbal information processing (Kutas and Federmeier, 2011). However, in associative learning cue stimuli images also feature clear semantic load signifying a demanded response (Guida et al., 2025). N400 latency may reflect the temporal dynamics of semantic or associative processing. Previous studies have shown that increasing familiarity or repetition can be accompanied by earlier N400 responses, consistent with more rapid access to relevant semantic or associative representations. (Renoult et al., 2012). More negative N400 amplitudes are commonly observed when a stimulus is less expected or receives less facilitation from the preceding semantic or associative context (Kutas and Federmeier, 2011). Accordingly, longer N400 latency as well as greater negative amplitude in the present task may reflect slower, more effortful or more prolonged processing of the learned cue–response association. Decrease in the latency and negative amplitude may indicate behavioral or neural adaptation leading to more efficient task performance.

It is known that the GMV exhibit reductions with age during the course of adolescence and young adulthood (Narvacan et al., 2017). In our study, this relationship is reversed by inclusion of the total intracranial volume as a covariate, demonstrating that while global GMV drops with age, clusters within pre-selected OFC and striatal ROIs lose gray matter slower that brain in general. Nevertheless, the normal developmental trajectory assumes decrease in the GMV even in these areas and, after accounting both for both age and TIV, smaller residual GMV estimates might be linked to more profound brain maturation processes, while larger to accelerated maturation. Therefore, both larger anterior N400 latency and negative amplitude from one side and greater striatum and sgACC volumes might suggest less mature neural substrate for associative learning function. Given age and TIV were included in the model as a covariate, this link is unlikely driven by global patterns of brain structural development with age. Instead, faster or slower brain maturation, relative to a participant’s chronological age and overall brain structural development trajectory, might contribute to both ERP and local GMV measures.

### 4.3 Neural correlates of P300 in associative learning

The P300 is commonly interpreted as an index of attentional resource allocation, evaluation of motivationally relevant outcomes, and updating of the current stimulus–context representation in working memory (Polich, 2007). At the Late stage of learning, females showed positive associations between anterior P300 amplitude to feedback events and GMV in the right NAcc/putamen and the right MOFC. However, this pattern was not found in males, and hence, it suggests a sex difference (descriptive in case of basal ganglia and statistically significant in case of MOFC) in the relationships between the Late stage feedback-processing related P300 amplitude and GMV in the frontostriatal reward pathway. These areas are typically functionally related to attention to motivationally salient feedback, that naturally occurs during the feedback presentation events. Specifically, the NAcc and putamen are implicated in reward-related learning, motivational salience, and the selection or reinforcement of actions that lead to favorable outcomes (Leng and Zhou, 2010; Balleine and O'Doherty, 2010). Interestingly, the GMV within the putamen though left-hemispheric is involved in another, N400-related structure-function relationship in the female sample, as mentioned above. The MOFC contributes to representing outcome value and flexibly using value information to guide future choices (Padoa-Schioppa and Cai, 2011). Thus, individual differences in the neural processing of feedback may be related to the structural organization of the frontostriatal system supporting reward evaluation and action–outcome learning based on the information provided with the feedback (Bromberg-Martin et al., 2010), at least in females, and sex differences are possible related to P300-MOFC function structure relationships.

In females, feedback-locked anterior P300 latency on the Late stage was negatively associated with GMV in the left sgACC. This result was not reproduced in the male sample, while sex differences were not significant. The ventral cingulate region is involved in affective and motivational evaluation of reward-related information (Rudebeck et al., 2014), expected while processing of the feedback on the trial correctness.

In males, anterior P300 amplitude to Early stage feedback provision was positively associated with GMV in the left hippocampus and left amygdala. The hippocampus contributes to the formation of cue–outcome associations, whereas the amygdala supports the processing of motivationally salient feedback and can modulate attention and memory for outcomes (Foerde et al., 2013; Fernando et al., 2013; Roesler et al., 2021).

As for N400, for P300 larger ERP amplitude typically reflects greater task complexity or increased cognitive effort, which may indicate less efficient executive control (Polich, 2007; Verleger, 2020). Thus, larger P300 amplitudes as well as greater GMV in the left amygdala/hippocampus and basal ganglia margins extended into conventional white matter areas for males as well as basal ganglia and MOFC GMV in females may serve as proxies for slightly delayed development of neural underpinnings of executive functions supporting associative learning. The sgACC negative link with the P300 latency is an outlying result, that cannot be explain by maturation alone and warranting specific subsequent research.

Taken together, the findings in female and male groups may suggest partially different structural correlates of feedback processing across learning stages. In females, the Late-stage associations involved striatal and OFC regions typically implicated in reward valuation and reinforcement of established action–outcome contingencies. In males, the early-learning association involved hippocampal–amygdala regions that may contribute to encoding salient outcome information while cue–response associations are still emerging. However, except for the right MOFC cluster result, this pattern should not be interpreted as evidence of a definitive sex difference, because the remaining differences between correlation coefficients were not statistically significant. All associations require replication in larger samples featuring larger power to test sex-by-brain-ERP interactions directly.

Several limitations should be acknowledged. First, the modest sample size in sex-stratified analyses might limit statistical power and prevent detection of weak effects. Second, the VBM method provides only indirect measures of brain structure and should be interpreted with caution in terms of tissue volumes. Finally, sex-specific findings revealed in the present study cannot be unequivocally attributed to sole biological or experiential determinants.

## 5 Conclusion

In summary, this ERP–VBM study found that, after accounting for age and TIV, N400 and P300 measures collected during associative learning task were related to regional GMV in participants aged 7–21 years. In females, onset-locked anterior N400 latency was associated with GMV in the left putamen/lentiform nucleus and subgenual ACC, while feedback-related anterior P300 amplitude was associated with GMV in the right NAcc/putamen and MOFC. In males, anterior N400 peak magnitude to Late onset events was associated with GMV in the caudate nucleus. The anterior P300 amplitude to feedback on the Early task stage was associated with GMV in the left hippocampus/amygdala cluster. These findings may suggest the relationships between N400- and P300-related processes and structural variation in striatal, limbic, and frontal regions, mostly aligning with neural maturation manifesting both structurally and functionally. The majority of sex differences in correlation coefficients were not statistically significant. However, the female group presented stronger ERP-VBM correlations for Late-stage onset-locked anterior N400 latency with the left ACC GMV and Late-stage feedback-locked anterior P300 amplitude with the right MOFC GMV.

## Data Availability

The datasets presented in this article are not readily available because the dataset is subject to privacy and personal-data restrictions imposed by the ethics committee. It may only be used for approved research purposes, and may not be shared or redistributed. Requests to access the datasets should be directed to almkusto@kaznu.kz.
